# Drug Tolerance to EGFR Tyrosine Kinase Inhibitors in Lung Cancers with *EGFR* Mutations

**DOI:** 10.3390/cells10071590

**Published:** 2021-06-24

**Authors:** Kenichi Suda, Tetsuya Mitsudomi

**Affiliations:** Division of Thoracic Surgery, Department of Surgery, Kindai University Faculty of Medicine, Osaka-Sayama 589-8511, Japan; mitsudom@med.kindai.ac.jp

**Keywords:** non-small cell lung cancer, drug-tolerant cells (DTCs), drug-tolerant persisters (DTPs), bypass pathway, acquired resistance, EGFR tyrosine kinase inhibitors (EGFR TKIs)

## Abstract

Epidermal growth factor receptor (EGFR) tyrosine kinase inhibitors (TKIs) serve as the standard of care for the first-line treatment of patients with lung cancers with *EGFR*-activating mutations. However, the acquisition of resistance to EGFR TKIs is almost inevitable, with extremely rare exceptions, and drug-tolerant cells (DTCs) that demonstrate reversible drug insensitivity and that survive the early phase of TKI exposure are hypothesized to be an important source of cancer cells that eventually acquire irreversible resistance. Numerous studies on the molecular mechanisms of drug tolerance of *EGFR*-mutated lung cancers employ lung cancer cell lines as models. Here, we reviewed these studies to generally describe the features, potential origins, and candidate molecular mechanisms of DTCs. The rapid development of an optimal treatment for *EGFR*-mutated lung cancer will require a better understanding of the underlying molecular mechanisms of the drug insensitivity of DTCs.

## 1. Introduction

Genotype-directed molecular-targeted therapies are the standard of care for a subset of lung cancers that harbor an activated oncogene with a driver mutation [1]. Epidermal growth factor receptor (*EGFR*) mutations are the most common driver gene mutations in lung cancers and are found in approximately 50% of lung adenocarcinomas in East Asians and in approximately 15% in Caucasians [2]. EGFR tyrosine kinase inhibitors (TKIs) such as first-generation gefitinib and erlotinib, second-generation afatinib and dacomitinib, and third-generation osimertinib are available in clinical practice for patients with *EGFR*-sensitizing mutations, which account for ≈90% of all *EGFR*-activating mutations. These EGFR TKIs are administered as monotherapy or, in the case of erlotinib or gefitinib, in combination with a fully humanized anti-VEGFR monoclonal antibody (ramucirumab).

Despite dramatic initial responses to EGFR TKIs, acquired resistance is almost inevitable [3], with extremely rare exceptions, after a median progression-free survival (PFS) of 9.2–14.7 months for first- or second-generation EGFR TKIs [4,5,6,7] and 18.9–19.4 months for osimertinib or a ramucirumab and erlotinib combination [8,9]. After the first identification of the mechanism of acquired resistance to EGFR TKI (gefitinib) [10], a secondary T790M mutation appears that substitutes a threonine residue with a methionine at codon 790 of *EGFR* exon 20, numerous efforts attempted to identify additional acquired resistance mechanisms to EGFR TKIs [11].

Second-line treatments that target an acquired resistance mechanism are reasonable treatment strategies to further improve patient outcomes, as exemplified by osimertinib being administered to patients with tumors with acquired resistance to first- or second-generation EGFR TKIs conferred by the T790M secondary mutation [12]. However, in vitro as well as clinical studies have demonstrated that a number of mechanisms of acquired resistance can arise after EGFR TKI treatment failure [11]; therefore, it is impractical to analyze all of these mechanisms in routine clinical practice in order to select an appropriate second-line treatment. In addition, our previous experiments using the HCC827 lung adenocarcinoma cell line (carrying the *EGFR* exon 19 deletion mutation, del E746_A750) indicate that cancer cells are flexible enough to always find a way to survive [13,14]. Effective front-line treatment strategies, such as the “primary double-strike therapy” we proposed in our previous review [3], for patients with *EGFR*-mutated lung cancers are therefore urgently required.

## 2. Incomplete Response to EGFR TKIs and the Concept of Drug-Tolerant Cells

### 2.1. Cancer Cells Always Survive after Exposure to EGFR TKIs in Clinical Settings

**Figure 1:**
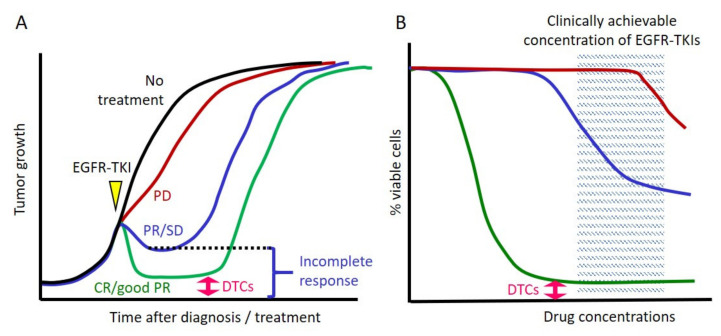
Clinical responses to EGFR TKIs and their corresponding in vitro growth-inhibitory curves. (**A**) *EGFR*-mutated lung cancer patients demonstrate different responses to an EGFR TKI as follows: complete response (CR), partial response (PR), stable disease (SD), and progressive disease (PD). Even in patients who experience a CR or a relatively strong PR, disease recurrence with the acquisition of resistance is inevitable; therefore, it is hypothesized that a small number of viable cells (drug-tolerant cells (DTCs)) remain during the period of maximum response. The yellow triangle indicates the initiation of EGFR TKI therapy. (**B**) In vitro cell line models are useful to mimic clinical responses to EGFR TKIs. Green: cell lines with the highest sensitivities; blue: those with moderate sensitivities; and red: those with inherent resistance. Each line of the growth-inhibitory curve corresponds to the clinical response drawn in the same color in Figure 1A. A small fraction of surviving cells remains even in cell lines with the highest sensitivity to EGFR TKIs (green line), and these remaining cells are often used as an in vitro model of DTCs.

In clinical practice, responses to EGFR TKIs are heterogeneous, including a complete response to the progressive disease, according to the Response Evaluation Criteria in Solid Tumors criteria (Figure 1A). Moreover, only a small fraction of patients with lung cancer with *EGFR* mutations (<5%) experience a complete response [4,5,6,7,8], although the *EGFR* mutation is a truncal mutation and is homogeneously distributed (i.e., virtually all tumor cells harbor the same *EGFR* mutation) [15,16,17]. Furthermore, we observe in our daily clinical practice that almost all patients eventually progress, including those who experience a complete remission. These facts indicate that some cancer cells are still viable after exposure to EGFR TKIs.

### 2.2. Cell Line Models That Mimic Clinical Responses to EGFR TKIs

These above clinical observations are modeled using lung cancer cell lines with *EGFR* mutations (Figure 1B). For example, in cell growth inhibitory assays using the first-generation EGFR TKI gefitinib, the cell lines NCI-H3255 (L858R), HCC827, HCC4006 (del L747_A750 ins P), and PC9 (del E746_A750) exhibit the highest sensitivities (Figure 1B), followed by PC3 (del L747_A750 ins P) and HCC2279 (del E746_A750) cells with moderate sensitivities. Additionally, H1975 (L858R/T790M) and H1650 (del E746_A750/*PTEN* null) cells exhibit inherent resistance [18]. Models using such cell lines are considered adequate for studying resistance to EGFR TKIs [19] because many of their resistance mechanisms recapitulate those identified in studies of clinical specimens obtained from TKI-refractory patients.

### 2.3. Inherent EGFR TKI Insensitivity in Cell Line Models

It is reasonable that cancer cells with de novo resistant mechanisms (such as the T790M mutation in H1975 cells and the *PTEN* null in H1650 cells) exhibit inherent resistance to EGFR TKIs. These cell lines may correspond to tumors that indicate progressive disease despite the presence of an activating *EGFR* mutation. Furthermore, decreased apoptosis upon exposure to EGFR TKIs can be partly explained by *BIM* deletion polymorphism [20] (as seen in PC3 and HCC2279 cells [18]), and this would be considered one of reasons for a clinical incomplete response to EGFR TKIs (Figure 1A).

### 2.4. The Concept of DTCs

In cell line models with the highest sensitivities to EGFR TKIs, the vast majority of *EGFR*-mutated lung cancer cells are killed within a few days upon exposure to a clinically relevant concentration of an EGFR TKI, whereas a small fraction of viable, largely quiescent cells (≈0.3%) remains detectable after several days in the presence of an EGFR TKI [21]. Sharma et al. were the first to analyze the details of these surviving cells, which they called drug-tolerant persisters (DTPs), in *EGFR*-mutated lung cancers [21]. Here, we refer to the surviving cells as drug-tolerant cells (DTCs) because this term is frequently encountered in the relevant literature. As exemplified by the growth-inhibitory curve (Figure 1B), this fraction of surviving cells usually reaches a plateau and will not be eradicated by increasing concentrations of EGFR TKI. An important feature of these surviving cells is the reversible nature of their drug-tolerant state [21], in which cells propagated in drug-free media rapidly reacquire EGFR TKI sensitivity. Therefore, we understand that DTCs can also be derived from single-cell cloned parental cells that do not harbor innate aberration(s) that confer insensitivity to TKIs. However, after long-term exposure, DTCs are hypothesized to be an important source of cancer cells that eventually acquire irreversible resistance mechanisms to EGFR TKIs [22,23]. Thus, from a therapeutic perspective, understanding the molecular mechanism(s) of DTCs is critical to prevent disease recurrence in patients who experience major or complete radiological responses. In terms of the reversibility of drug insensitivity, DTCs should be clearly distinguished from minor resistant sub-clones that may exist prior to treatment [3]. As well demonstrated by Hata, et al., acquired resistance can result from either the acquisition of an irreversible resistance mechanism by DTCs or the selection of pre-existing minor resistant sub-clones [22].

## 3. Features of DTCs

### 3.1. How Are DTCs Induced upon Exposure to an EGFR TKI?

**Figure 2:**
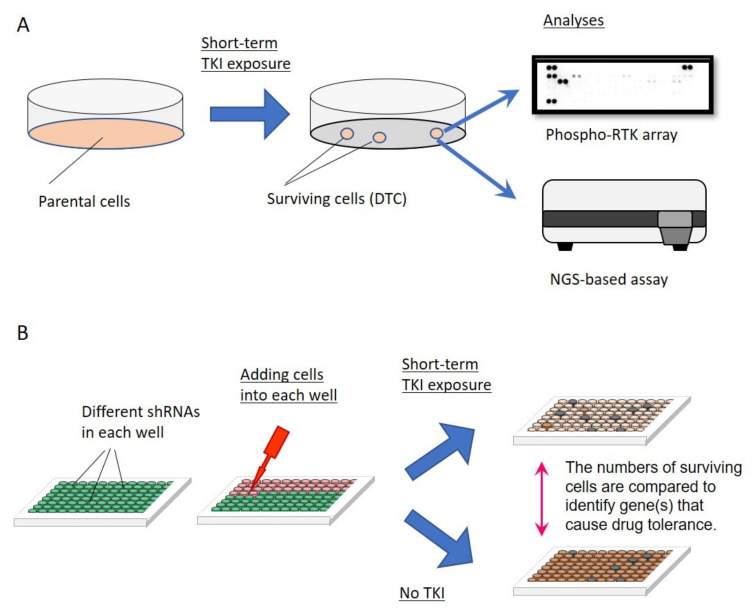
Strategies to explore the molecular mechanisms of drug-tolerant cells (DTCs). (**A**) *EGFR*-mutated lung cancer cell lines are subjected to short-term treatment with an EGFR TKI at clinically equivalent concentration(s). The remaining cells are collected and comprehensive analyses are performed to identify activated molecules/signaling pathways in the presence of EGFR TKIs. (**B**) shRNA-, siRNA-, or CRISPR/Cas9-mediated screening is performed to search for molecules that reduce cell survival specifically in the presence of an EGFR TKI when target expression is inhibited.

Numerous studies on DTCs in *EGFR*-mutated lung cancers that followed the discovery of Sharma et al. [21] used short-term treatment with an EGFR TKI at clinically achievable concentrations to establish DTCs against EGFR TKIs (Figure 2A). As summarized in Section 4, these studies have identified multiple molecular mechanisms of drug tolerance [22,24,25,26,27,28,29,30,31,32,33,34,35,36,37,38,39,40,41,42,43,44,45], although they do not identify the original cells that can become DTCs while the majority of cells are killed. These studies also do not answer the question of how these few cells can acquire specific molecular mechanism(s) of drug tolerance.

**Figure 3:**
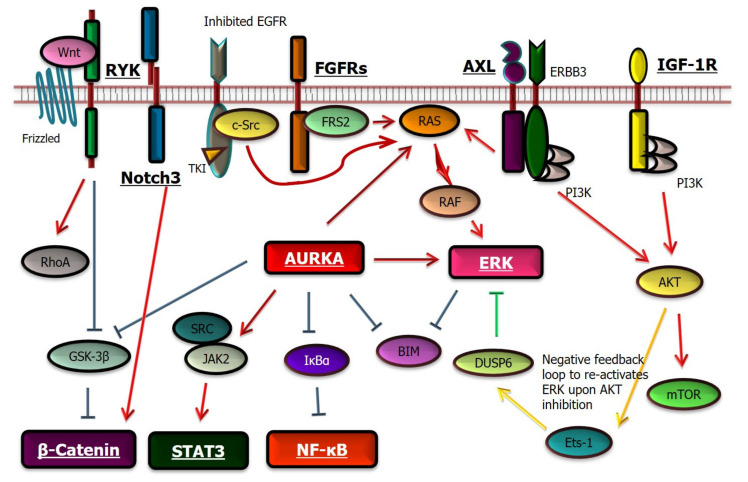
Molecules and associated signaling pathways that may mediate drug tolerance in *EGFR*-mutated lung cancer cells upon treatment with an EGFR TKI. Molecules known to cause drug tolerance are indicated with underlined bold letters. Molecules such as PI3K, AKT, mTOR, or c-Src may play important roles in drug tolerance [36,52], and other candidates not illustrated here include inhibited apoptosis, altered chromatin state, stabilized EGFR through a de-ubiquitinase, involvement of the tricarboxylic acid (TCA) cycle, induced ER stress, and upregulation of cholesterol synthesis.

Evidence indicates that the inhibition of EGFR activity triggers the switch that induces DTCs, such as the inactivation of AKT/Ets-1 signaling [31]. Ets-1 inactivation inhibits the transactivation of its target genes (cyclins D1, D3, and E2), and cells become quiescent. Furthermore, Ets-1 inactivation inhibits the transcription of dual specificity phosphatase 6 (DUSP6), a negative regulator of ERK1/2, thereby reactivating ERK1/2 and contributing to the ability of *EGFR*-mutated lung cancer cells to maintain proliferation and survival signals (please see Figure 3). In addition, the expression of branched-chain amino acid aminotransferase 1 (BCAT1), induced by inhibited EGFR activity, reportedly promotes insensitivity to TKIs by attenuating the accumulation of reactive oxygen species [40]. However, these findings are insufficient to explain why only a small fraction of cells acquire the DTC phenotype.

Epigenetic mechanisms may be responsible for the induction of DTCs because of the reversible nature of drug tolerance. For example, demethylation of H3K4 or methylation H3K9 and H3K27 are associated with tolerance to EGFR TKIs [21,46]. However, it is unknown how the inhibition of EGFR activity induces these epigenetic changes only in a small fraction of *EGFR*-mutated lung cancer cells.

### 3.2. Which Cells Will Become DTCs upon Exposure to EGFR TKIs?

Another potential answer to the question of the origin of DTCs is provided by a study of *BRAF* V600E-mutated melanoma cell line models treated with vemurafenib [47]. Long-term time-lapse imaging demonstrates that drug-tolerant colonies arise from normally proliferating single cells before drug addition, indicating that these cells are not in a dormant state before TKI exposure. High-throughput single-molecule RNA FISH analysis reveals that certain populations of rare cells (frequencies, 1:50–1:500) express high levels of mRNAs such as *EGFR*, *AXL*, or *WNT5A* that can contribute to drug resistance, and these rare cells are more likely to become tolerant in the presence of the drug [47]. However, such a resistance state is not heritable. For example, *EGFR*-high expressing cells, collected using flow cytometry, returns to the normal level of *EGFR* expression in a few weeks [47]. In this study, one lung adenocarcinoma cell line (PC9) in addition to four melanoma cell lines were tested, and rare populations of PC9 cells express high *PDGFRB* or *FOSL1*. This means that the similar profound transcriptional variability of a cell may predict cells among the population of *EGFR*-mutated lung cancer cells that will ultimately become tolerant to EGFR TKIs.

### 3.3. Establishment of DTCs and Optimizing the Concentrations of EGFR TKIs

DTCs have been established using different EGFR TKIs, drug concentrations (1–2000 nM), and times of exposures (24 h to 3 weeks). However, few studies evaluated the effects of drug concentrations [40,45] or times of exposures [31,36,37,44] on the induction of DTCs.

Regarding the drug concentrations, using PC9 cells and gefitinib (clinically achievable concentration: approximately 800 nM), Wang et al. [40] determined the effects of pretreatment with gefitinib at sub-lethal concentrations for 2 h before administering a lethal concentration. The drug tolerance effect was highest when cells were exposed to 50 nM gefitinib [40] and TKI tolerance was maintained for >2 h after drug withdrawal and diminished gradually for approximately 6 h [40]. Higher doses of gefitinib (100–1000 nM) failed to induce drug tolerance during this short exposure schedule, suggesting increased cytotoxicity of pretreatment at such high concentrations [40]. These findings were confirmed using the HCC827 and SH450 cell lines and in erlotinib pretreatment experiments [40].

Longer exposure of HCC4006 cells to EGFR TKIs (72 h) causes the opposite effect in our recent study [45]. Specifically, clinically achievable concentrations of osimertinib (600 nM), but not afatinib (60 nM), induced the drug-tolerant phenotype in HCC4006 cells. Phosphorylation of EGFR was completely inhibited in the presence of 600 nM osimertinib but was partially retained in the presence of 60 nM afatinib [45]. When we examined the effects of drug concentrations on the inducibility of the DTC phenotype, we found that higher concentrations of afatinib (>180 nM) induced the DTC phenotype and that lower concentrations of osimertinib (<200 nM) failed to induce the DTC phenotype, indicating that sufficient inhibition of EGFR phosphorylation was required to induce the DTCs [45]. PC9 and H1975 cells were similarly affected. These findings are consistent with the results of a previous study demonstrating ERK1/2 reactivation as a molecular mechanism of DTCs caused by negative feedback of AKT inhibition upon gefitinib exposure [31]. Specifically, the magnitudes of AKT inhibition and ERK1/2 reactivation are dependent on the dose of gefitinib [31].

Regarding the times of exposure, studies illustrate that 24 h [31,37] or 48–72 h [36,44] are sufficient to induce activation of molecules that cause drug tolerance after initiation of exposure to EGFR TKIs.

### 3.4. “Preference” for Drug Tolerance Mechanisms of Each Cell Line

In 2012, we summarized a review article that reported that each *EGFR*-mutated lung cancer cell line may employ a “preferred” mechanism upon acquisition of resistance to EGFR TKIs [11] (e.g., *MET* gene amplification in HCC827 cells and the induction of the epithelial-to-mesenchymal transition (EMT) phenotype in HCC4006 cells). Therefore, it is not surprising that each *EGFR*-mutated lung cancer cell line employs their “preferred” mechanism(s) to achieve drug tolerance. For example, in the aforementioned study of melanoma cell lines [47], sporadically elevated expressions of markers of resistance to vemurafenib were detected among different melanoma cell lines as follows: *EGFR* in WM986-A6 and 1205Lu cells, *AXL* in WM986-A6 and WM983B-E9 cells, and *FOSL1* in SK-MEL-28 and 1205Lu cells [47].

Among studies of DTCs in *EGFR*-mutated lung cancers, a few suggested a possible “preference” for drug tolerance mechanism(s) in cell lines [37,44]. Specifically, in *EGFR*-mutated lung cancer cell lines expressing high levels of AXL (PC9 and HCC4011 cells), a small population of tumor cells tolerant to osimertinib emerged as persisters by restoring the survival signal generated by AXL [37]. In contrast, in *EGFR*-mutated lung cancer cell lines expressing low levels of AXL (HCC827, HCC4006, and H3255 cells), EGFR TKI tolerance was mediated by an insulin-like growth factor-1 receptor (IGF-1R) through the induction of its transcription factor FOXA1 [44].

### 3.5. Diversity of Molecular Mechanisms That Mediate Drug Tolerance

While cancer cells may have a “preference” for drug tolerance mechanisms, it is also true that multiple drug tolerance mechanisms have been reported in each lung cancer cell line. For example, >10 drug tolerance mechanisms (involving IGF-1R, AXL, FGFR3, AURKA, STAT3, NF-kB, YAP/TEAD, and other mechanisms) are employed by PC9 cells [28,33,34,35,36,37,38,39,42,43,44]. This diversity may be partly explained by the properties of EGFR TKIs, differences in their concentrations, differences in TKI exposure times, or combinations of these experimental manipulations. However, the ability of a single cell line to develop multiple drug tolerance mechanisms strongly suggests that eradicating all cancer cells by co-targeting a single drug tolerance mechanism will be a formidable task [3].

A few studies suggest that different populations of DTCs may emerge at the same time during exposure to an EGFR TKI in a single dish. Using PC9 cells as a model of DTCs, Kunimasa et al. observed two types of DTCs after exposure to 2 µM gefitinib as follows: (1) a CD133^high^ cell population with cancer stem cell (CSC) properties and (2) a CD133^low^ cell population with features of therapy-induced senescence [32]. Senescent cells communicate with neighboring cells through numerous secretory factors such as inflammatory cytokines, chemokines, and growth factors (senescence-associated secretory phenotype (SASP)) [48]. Evaluation of the relationship between CD133^low^ and CD133^high^ DTCs revealed that the CD133^low^ cell population supports the emergence of the CD133^high^ cell population through the SASP. Furthermore, in another study using PC9 cells as a model of DTCs, YAP-negative (60%) and YAP^high^ (40%) cell populations remained after a 10-day treatment with 100 nM osimertinib [42]. The YAP-negative cells underwent ERK1/2 reactivation that conferred drug tolerance, while YAP^high^ cells exhibited senescence-like dormancy through the YAP/TEAD-mediated transcriptional reprogramming of the apoptotic pathway [42]. Additionally, in a recent study, PC9-derived DTCs were traced using a novel “watermelon system” comprising a high-complexity, barcoded lentiviral library designed to simultaneously trace each cell’s clonal origin, proliferative state, and transcriptional state [49]. This study demonstrates that cycling and non-cycling DTCs arise from different pre-existing cell lineages with distinct transcriptional and metabolic programs [49]. Moreover, these cycling DTCs express upregulated antioxidant gene programs and undergo a metabolic shift to fatty acid oxidation.

## 4. Summary of Molecular Mechanisms Conferring Drug Tolerance in *EGFR*-Mutated Lung Cancer Cell Lines

### 4.1. Search Criteria for Published Studies

To identify published articles that analyzed DTCs and their molecular mechanisms, we systematically searched PubMed for relevant studies as of 2 December 2020. Our search criteria included the following terms: “drug tolerance” or “drug tolerant”, “lung cancer,” and “EGFR”. We manually scanned the reference lists of select articles for additional eligible publications. We finally identified 23 relevant papers that report potential mechanisms of drug tolerance [21,22,24,25,26,27,28,29,30,31,33,34,35,36,37,38,39,41,42,43,44,50,51]. We included our recent paper describing the generation of DTCs from multiple *EGFR*-mutated lung cancer cell lines [45]. Each study used *EGFR*-mutated lung cancer cell lines to identify candidate essential molecule(s)/pathway(s) for DTC induction by establishing DTCs via short-term exposure to EGFR TKIs (Figure 2A) or by using shRNA-, siRNA-, or CRISPR/Cas9-mediated screening (Figure 2B).

### 4.2. Mechanisms of Drug Tolerance—Activation of Bypass Signaling

Activation of other proto-oncogenes is a common mechanism of acquired resistance to EGFR TKIs [11]. Furthermore, the aforementioned study of melanoma cell lines found that rare cells express resistance genes (e.g., *EGFR*, *AXL*, or *WNT5A*) at high levels and that these cells are far more likely to become tolerant once a drug is applied [47]. These findings support the hypothesis that the activation of bypass signaling may play important roles in the acquisition of the drug-tolerant phenotype. However, in contrast to mechanisms of acquired resistance to EGFR TKIs, these bypass signaling activations are not associated with genetic changes because drug tolerance is reversible.

Among 24 studies that reported the molecular mechanisms of drug tolerance, 18 focused on the activation of bypass signaling (Figure 3) [21,22,24,25,26,27,28,29,30,31,35,36,37,38,44,45,50,51]. Subsequent to the first report of IGF-1R activation in 2010 [21], AXL [37], Notch3 [50], and fibroblast growth factor receptor 3 (FGFR3) [38] are identified as receptor tyrosine kinases (RTKs) that cause drug tolerance in lung cancers with *EGFR* mutations. In the latter study, upregulation of FGFR3 expression together with increased expression of multiple FGF ligands was identified through analyses of HCC827, PC9, and H1975 cells [38]. However, in analyses of HCC4006 cells, two independent studies [44,45] observed that FGFR3 phosphorylation increases after exposure to osimertinib, although further analyses revealed that FGFR3 activation does not contribute to the molecular mechanism of drug tolerance. Moreover, we recently reported a potential role of receptor-like tyrosine kinase (RYK) in drug tolerance [45]. RYK binds with WNT to activate the canonical and noncanonical WNT pathways. Western blotting in some studies illustrates that, even when the alternative RTK pathway is activated in DTCs, the expression level of EGFR itself does not change significantly [36,38,42,44].

Numerous intracellular molecules are also candidate inducers of drug tolerance in *EGFR*-mutated lung cancers. These include ERK [31], Aurora kinase A (AURKA) [36], STAT3 [26,28], NF-kB [24,25,29,30,35], and β-catenin [27,51]. These results suggest that *EGFR*-mutated lung cancer cells may possess multiple molecules that can mediate survival during the early phase of EGFR TKI exposure at a lethal concentration. However, targeting a “master key” of these pathways (e.g., AURKA or ERK, Figure 3) may significantly contribute to the eradication of DTCs.

### 4.3. Mechanisms of Drug Tolerance—Dysregulation of the Apoptotic and Other Pathways

Another candidate mechanism of drug tolerance to EGFR TKIs involves increased YAP/TEAD activity [42] that engages the EMT transcription factor, SLUG, to directly repress pro-apoptotic BMF and limit drug-induced apoptosis. Furthermore, increased synthesis of MCL-1 serves as a molecular mechanism of drug tolerance via suppression of apoptosis [33].

Other potential mechanisms are diverse. For example, the de-ubiquitinase USP13 was identified through an siRNA screen (Figure 2B) of libraries comprising genes associated with the ubiquitin and ubiquitin-like cellular processes [43]. USP13 specifically counteracts the downregulation of mutated EGFR through the activities of ubiquitin ligases to cause drug tolerance. UFMylation, a recently identified ubiquitin-like modification, contributes to drug tolerance to erlotinib plus THZ1 (a CDK7/12 inhibitor) [34], a combination that suppresses DTCs [53]. Furthermore, the absence of UFMylation induces ER stress, which then enhances the induction of STING to promote pro-tumorigenic inflammatory signaling [34]. These findings support the conclusion that ER stress signaling promotes the survival of DTCs [34].

Another study of DTCs treated with osimertinib observed dysfunction of the TCA cycle and a pseudohypoxic response, which is mediated by hypoxia-associated proteins, independent of oxygen status [39]. The repression of Von Hippel–Lindau (VHL) disease by miR-147b and succinate dehydrogenase contributes to these processes [39]. Furthermore, upregulated expression of cytochrome P450 (CYP51A1) in DTCs is directly involved in cholesterol synthesis [41], and the CYP51A1 inhibitor, ketoconazole, downregulates cholesterol synthesis and overcomes the emergence of EGFR TKI tolerance.

## 5. Summary

In this paper, we summarized current understandings of DTCs that counteract the cytotoxic effects of EGFR TKIs in lung cancers that harbor an *EGFR* mutation. It is difficult to obtain clinical specimens that contain such DTCs because re-biopsy of a smaller tumor after initial TKI treatment is challenging. Therefore, research on cell lines that reflects the phenotypes of their cognate primary cancer cells is important to advance the treatment of lung cancers that express a constitutively activated EGFR. However, the diverse mechanisms of drug tolerance reported here can be employed by single cell lines. Further studies are therefore required to fully understand molecular mechanisms of drug tolerance to EGFR TKIs, which will contribute to the efforts to develop clinically relevant treatment strategies that co-target DTCs.

## Data Availability

The data presented in this study are available on request from the corresponding author.
